# Visual field defects of optic neuritis in neuromyelitis optica compared with multiple sclerosis

**DOI:** 10.1186/1471-2377-10-45

**Published:** 2010-06-18

**Authors:** Hideto Nakajima, Takafumi Hosokawa, Masakazu Sugino, Fumiharu Kimura, Jun Sugasawa, Toshiaki Hanafusa, Toshiyuki Takahashi

**Affiliations:** 1 Department of Internal Medicine I, Osaka Medical College, Takatsuki, Osaka, Japan; 2 Department of Internal Medicine, Seikeikai Hospital, Sakai, Osaka, Japan; 3 Department of Ophthalmology, Osaka Medical College, Takatsuki, Osaka, Japan; 4 Department of Neurology, Tohoku University Graduate School of Medicine, Sendai, Miyagi, Japan

## Abstract

**Background:**

Neuromyelitis optica (NMO) is an inflammatory demyelinating disease that predominantly affects the optic nerves and the spinal cord, and is possibly mediated by an immune mechanism distinct from that of multiple sclerosis (MS). Central scotoma is recognized as a characteristic visual field defect pattern of optic neuritis (ON), however, the differing pathogenic mechanisms of NMO and MS may result in different patterns of visual field defects for ON.

**Methods:**

Medical records of 15 patients with NMO and 20 patients with MS having ON were retrospectively analyzed. A thorough systemic and neurological examination was performed for evaluating ON. The total number of relapses of ON and visual fields was investigated. Visual fields were obtained by Goldmann perimeter with each ON relapse.

**Results:**

All MS patients experienced central scotoma, with 90% of them showing central scotoma with every ON relapse. However, 53% of NMO patients showed central scotoma with every ON relapse (p = 0.022), and the remaining 47% of patients experienced non-central scotoma (altitudinal, quadrant, three quadrant, hemianopia, and bitemporal hemianopia). Thirteen percent of NMO patients did not experience central scotoma during their disease course. Altitudinal hemianopia was the most frequent non-central scotoma pattern in NMO.

**Conclusions:**

NMO patients showed higher incidence of non-central scotoma than MS, and altitudinal hemianopia may be characteristic of ON occurring in NMO. As altitudinal hemianopia is highly characteristic of ischemic optic neuropathy, we suggest that an ischemic mechanism mediated by anti-aquaporin-4 antibody may play a role in ON in NMO patients.

## Background

Neuromyelitis optica (NMO; Devic's disease) is an idiopathic inflammatory disease of the central nervous system (CNS) that mainly affects the optic nerve and spinal cord. Traditionally, NMO is believed to differ from multiple sclerosis (MS) by causing very severe, often bilateral, optic neuritis (ON) and longitudinally extensive MRI spinal cord lesions but no MRI brain lesions or aggressive progression to disability and death [1]. Recent studies have reported a high frequency of brain MRI abnormalities in NMO patients. However, most were nonspecific and were not considered typical of MS, and hypothalamic involvement has been emphasized [2]. NMO has a more negative outcome than MS, with frequent and early relapses. Within 5 years of onset, 50% of patients have become blind in both eyes and cannot walk unassisted, and 20% die of respiratory failure due to cervical myelitis [3]. Although no controlled therapeutic trials have been specifically performed in NMO, case series and observational studies suggest that azathioprine in combination with oral steroid reduces the frequency of attacks [4,5], and rituximab and plasmapheresis can induce clinical remission of NMO [6-8]. Immuno-suppression rather than interferon β is the preferred treatment. Thus, distinguishing NMO from MS is very important for the therapeutic strategy of these disorders. Recently, clinical, neuroimaging, laboratory, and pathological features have been established showing that NMO is distinct from MS. Histopathological and serological findings strongly suggest the involvement of the humoral immune system [9]. In particular, detection of serum anti-aquaporin-4 (AQP4) antibody can be used to distinguish NMO from MS [10,11]

ON is the most common and often initial symptom in both NMO and MS. In acute ON, the cardinal field defect is a widespread depression of sensitivity, and visual field testing typically reveals a central scotoma, although other visual field changes such as color blindness, bitemporal hemianopia, paracentral scotoma, and altitudinal deficits have also been reported. ON in NMO tends to be more severe and recovery is less complete compared with attacks of ON in the context of MS [3]. Clinical features such as ocular pain, visual field deficits, and positive phenomena, i.e. movement-induced phosphenes, have been thought not to differ from those found in MS-associated attacks [3]. Unlike patients with MS, those with NMO experience more severe disease symptoms due to myelitis characterized by centrally located spinal cord lesions that are longer than three vertebral segments and frequent early attacks. In NMO, the pathophysiology of spinal cord lesions and relation with seropositivity for anti-AQP4 antibody are well investigated [12,13]. However, the difference of clinical symptoms or pathophysiologic findings for ON between NMO and MS have rarely been evaluated. We hypothesized that the differing pathogenic mechanisms of NMO and MS may result in different patterns of visual field defects as findings of ON. In this study, we evaluated the features of visual field defects in patients with NMO.

## Method

We retrospectively analyzed the medical records of 15 patients with NMO (all women, mean age of onset: 36 ± 11, mean ± SD) and 20 patients with MS (5 men and 15 women, 29 ± 9), all of whom had ON. NMO patients fulfilled Wingerchuk's revised diagnostic criteria [14], except for NMO-IgG seropositive status. MS patients included in this study had definitive MS according to McDonald's criteria [15]. A thorough systemic and neurological examination was performed to evaluate ON. Visual field tests were performed on the Goldmann perimeter whenever visual acuity permitted. MRI was performed where deemed necessary and for those who could afford the investigation. This study received institutional review board approval and informed consent was obtained according to the Declaration of Helsinki.

Serum samples were stored at - 80°C until testing for anti-AQP4 antibody. Anti-AQP4 antibody was assessed as described previously [12,16]. Briefly, human embryonic kidney cells (HEK-293) were stably transfected with either a vector containing AQP4-cDNA or empty vector, and specimens were tested by indirect immunofluorescence using these two cell lines (with or without AQP4). Specimens were incubated with the cells for 1 h, washed in phosphate-buffered saline (PBS), incubated with Alexa Fluor 488 goat anti-human IgG (Invitrogen, Eugene, Oregon, USA) for 30 min, and washed in PBS. The cells were then fixed in 4% paraformaldehyde and mounted in Permafluor aqueous mounting media (Beckman Coulter, Marseille, France).

### Statistics

All data in this study are presented as mean ± SD. Categorical variables were compared using the Mann-Whitney's U test and the Fisher's exact probability test. Significance levels were set at *P *< 0.05.

## Results

A total of 35 patients were included in this study: 15 NMO and 20 MS (Table 1). There were no differences between the 2 groups in gender and disease duration. Patients with NMO were older at disease onset, exhibited an increased number of total and ON relapses, and had a higher expanded disability status scale (EDSS) score.

**Table 1:** Demography and ocular findings of NMO and MS patients

	NMO	MS	*P*
	(n = 15)	(n = 20)	value
Sex (male/female)	0/15	5/15	0.057
Age at onset	36.0 ± 10.9	29.2 ± 8.8	0.038
Duration of disease (years)	14.4 ± 8.8	11.5 ± 9.9	0.216
Number of total relapses	10.1 ± 4.8	6.3 ± 6.4	0.007
Number of ON relapses	3.4 ± 3.2	1.7 ± 0.8	0.004
EDSS score	5.1 ± 2.5	2.6 ± 1.6	0.009
Brain lesion, % (n)	60 (9)	90 (18)	0.051
Spinal lesion, % (n)	100 (15)	60 (12)	0.005
LESCL, % (n)	87 (13)	10 (2)	<0.001
Anti-AQP4 antibody, % (n)	100 (15 )	0	<0.001

NMO = neuromyelitis optica; MS = multiple sclerosis; ON = optic neuritis; EDSS = expanded disability status scale; LESCL = longitudinally extensive spinal cord lesions.

When comparing visual field defect patterns of ON between the 2 groups, central scotoma was present in 31 out of 33 ON episodes in MS (94%) and 39 out of 51 episodes in NMO (76%) (p = 0.041, Table 2). In 51 episodes of ON, NMO patients exhibited 12 episodes of non-central scotoma (24%). Of the visual field defect patterns other than central scotoma, NMO patients showed 5 for altitudinal, 3 for quadrant, 2 for three quadrant, 1 for hemianopia, and 1 for bitemporal hemianopia. MS patients showed 1 each for three quadrant and hemianopia (Table 2).

**Table 2:** Visual field defect patterns of NMO and MS patients

	NMO	MS	*P *value
Total number of ON relapses	51	33	
Visual field defects			
Central scotoma (%)	39 (76)	31 (94)	0.041
Non-central scotoma			
Altitudinal (%)	5 (10)	0 (0)	0.151
Quadrant (%)	3 (6)	0 (0)	0.276
Three quadrant (%)	2 (4)	1 (3)	1
Hemianopia (%)	1 (2)	1 (3)	1
Bitemporal hemianopia (%)	1 (2)	0 (0)	1

NMO = neuromyelitis optica; MS = multiple sclerosis; ON = optic neuritis.

**Figure 1:**
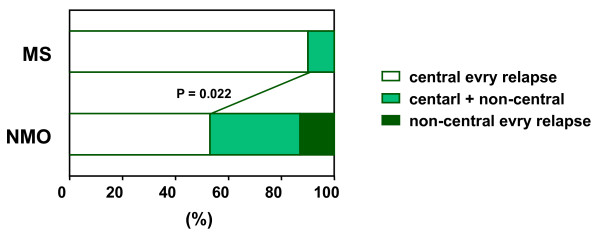
**Comparison of visual field defects during the disorders**. Ninety percent of MS patients showed central scotoma every time, but 54% in NMO (p = 0.022). In NMO patients, 33% of patients showed both central scotoma and non-central scotoma, and 13% of patients showed non-central scotoma every time. MS = multiple sclerosis; NMO = neuromyelitis optica.

During the course of the disease, 90% of MS patients (18/20) showed central scotoma with every episode; however, central scotoma with every episode was present in 54% of NMO patients (8/15) (p = 0.022, Figure 1). In the remaining 7 NMO patients, 5 showed both central and non-central scotoma, and 2 patients showed non-central scotoma with every episode. In 7 NMO patients showing non-central scotoma, altitudinal hemianopia was most frequent (5/7), and the location of the altitudinal hemianopia was inferior in 3 of 5 relapses and superior in 2 relapses. Notably, all altitudinal hemianopia occurred at the initial attack of each eye (Table 3).

**Table 3:** Clinical findings of 7 NMO patients with non-central scotoma

Patient No.	1	2	3	4	5	6	7
Sex/Age at onset	F/48	F/21	F/47	F/34	F/32	F/32	F/54
Duration of disease (years)	9	11	24	4	15	18	12
EDSS score	2.5	1	7	3.5	7	7.5	4
Number of total relapses	5	5	12	6	11	18	23
Number of ON relapses	3	2	2	2	3	4	12
Ocular pain	moderate	none	none	mild	mild	none	moderate
Optic disk in acute phase	normal	NE	NE	normal	normal	normal	normal
Course of ON	lt-altitudinal	lt-three quadrant	rt-altitudinal	lt-altitudinal	rt-altitudinal	bil-central	rt-central
	(inferior)	rt-altitudinal	(superior)	(inferior)	(superior)	lt-guadrant	lt-central: 2nd-3rd
	rt-central	(inferior)	rt-central	rt-quadrant	lt-central	rt-hemianopia	rt-quadrant
	lt-central				lt-central		lt-three quadrant
							rt-central
							lt-central
							rt-central: 8th-11th
							bitemporal
Outcome of ON	rt-recover	rt-recover	rt-recover	rt-recover	rt-light perception	rt-recover	rt-light perception
	lt-recover	lt-recover		lt-recover	lt-light perception	lt-recover	lt-light perception

NMO = neuromyelitis optica; EDSS = expanded disability status scale; ON = optic neuritis; NE = not evaluated.

## Discussion

A variety of visual field defects may be seen in optic neuropathies, including central, centrocecal, arcuate, altitudinal, and nasal step field defects. Central scotoma is recognized as a typical visual field defect pattern of ON in MS [17]. In this study, all MS patients experienced central scotoma, with 90% showing central scotoma with every ON attack. On the contrary, 53% of NMO patients showed central scotoma with every ON attack, and the remaining 47% of patients experienced non-central scotoma. Moreover, 13% of NMO patients did not experience central scotoma during the course of their disease. Of the non-central scotoma patterns, altitudinal hemianopia was most frequent. Since altitudinal hemianopia was not recognized in MS patients, this visual field defect may be characteristic of ON for patients with NMO.

ON is the initial manifestation of NMO in 77% of patients. In 30% of NMO patients, the initial attack of ON led to blindness, with only 43% of patients completely recovering from the first attack. Compared with MS patients, NMO patients had a significantly higher rate of bilateral ON (70% versus 19%) [18]. Although the optic nerve is mainly affected in both NMO and MS, the pathogenesis of ON in NMO might differ from that of MS. Compared to MS, the study using optical coherence tomography indicated a thinner overall average retinal nerve fiber layer, suggesting widespread axonal injury in the affected optic nerves in NMO [19].

Pathologically, NMO shares with MS a pattern of focal demyelination, inflammation, scar formation, and axonal destruction, but NMO also has an intense perivascular response, prominent necrosis, and cavitation, which are not seen in MS [9]. IgG, IgM, and products of complement activation are deposited in a perivascular pattern in NMO, suggesting a pathogenic role involving autoantibodies [9]. Blood vessels within demyelination spinal lesions of NMO are thickened and hyalinized [20]. Active lesions exhibit tissue swelling, infiltrating polymorphonuclear macrophages, active microglia, demyelination, axonal loss, prominent necrosis, and variable degrees of perivascular inflammation with prominent eosinophils and products of their exocytosis. Chronic lesions show gliosis, cystic degeneration, cavitation, and atrophy [21]. These findings suggest that a humoral effector mechanism is initiated by binding of the NMO antibody at the blood-brain barrier (BBB).

Several studies have reported that areas of CNS inflammation correlate with expression pattern of AQP4 in NMO [22,23]. Expression of AQP4 in the brain and spinal cord is associated with astrocyte membranes that appose endothelial cell basal membranes. Astrocytes interact extensively with endothelial cells to maintain the CNS BBB, which normally limits the access of immune system effectors unless localized or distant events disrupt the BBB, thus allowing access of cellular or soluble immune effectors. AQP4 is also expressed by astrocytes that surround the optic nerve [24]. Since the optic nerve head is an area of the CNS where the BBB is more permissive, as evidenced by immunostaining for markers of intact BBB [25,26], tissues of the optic nerve might be more sensitive to AQP4 dysfunction mediated by anti-AQP4 antibodies [27]. Thus, in NMO, optic nerve lesions would have demyelination, axonal loss, and perivascular response, as seen in spinal cord lesions.

Central scotoma is recognized to be a typical visual field defect pattern of ON in MS. In this study, NMO patients showed higher incidence of non-central scotoma than MS patients (p = 0.022, Figure 1); altitudinal hemianopia was more common in non-central scotoma. An altitudinal visual field defect is suggestive of ischemic optic neuropathy, which occasionally is the result of posterior ciliary artery occlusion [28,29]. We suggest that ischemic mechanism mediated by anti-AQP4 antibody may play a role in ON for NMO patients. Pathological study demonstrated that vascular degeneration, such as thickened or hyalinized vessels, existed in the spinal cord lesions [21]. Recent study indicated that NMO patients showed more vascular changes, including attenuation of the peripapillary vascular tree and focal arteriolar narrowing as the retinal features of ON than MS patients [30]. These vascular changes may results from direct vascular inflammation mediated by anti-AQP4 antibody [30,31]. Therefore, the tissue organization of optic nerve cells, such as the vascular structures associated with the optic nerves, is thought to express AQP4, resulting in non-central scotoma, especially altitudinal hemianopia.

Although NMO is often fulminant and has a more negative outcome than MS [32], NMO responds to glucocorticoids, immunosuppressive agents, or plasmapheresis. Since monosymptomatic ON is often seen as being the first indication of an attack of NMO and MS, ophthalmoscopic examination, especially the visual field test, is helpful for diagnosis of NMO, and anti-AQP4 antibody should be checked to decide the most effective treatment [33].

## Conclusion

NMO patients showed higher incidence of non-central scotoma than MS, and altitudinal hemianopia may be characteristic of ON occurring in NMO. As altitudinal hemianopia is highly characteristic of ischemic optic neuropathy, we suggest that an ischemic mechanism mediated by anti-aquaporin-4 antibody may play a role in ON in NMO patients.

## Competing interests

The authors declare that they have no competing interests.

## Authors' contributions

HN performed analyses, collected data and wrote the manuscript. TH helped to draft the manuscript and collected data. MS, FK, JS, and TH helped to draft the manuscript. TT performed anti-AQP4 antibody assay. All authors read and approved the final manuscript.

## Pre-publication history

The pre-publication history for this paper can be accessed here:

http://www.biomedcentral.com/1471-2377/10/45/prepub
